# Disease manifestations and burden of illness in patients with acid sphingomyelinase deficiency (ASMD)

**DOI:** 10.1186/s13023-017-0572-x

**Published:** 2017-02-23

**Authors:** Margaret M. McGovern, Ruzan Avetisyan, Bernd-Jan Sanson, Olivier Lidove

**Affiliations:** 1 Department of Pediatrics, Stony Brook University School of Medicine, Stony Brook, NY 11794 USA; 2 Sanofi Genzyme, Cambridge, MA USA; 3 Department of Internal Medicine-Rheumatology, Hôpital de la Croix Saint Simon, Paris, France; 4 Sorbonne Universités UPMC Univ Paris 06, INSERM, CNRS, Centre de Recherche en Myologie, GH Pitié Salpêtrière, Paris, France

**Keywords:** Acid sphingomyelinase deficiency, ASMD, Burden of illness, Disease manifestations, Lysosomal storage disorder, Natural history, Niemann-Pick disease types A and B

## Abstract

Acid sphingomyelinase deficiency (ASMD), a rare lysosomal storage disease, is an autosomal recessive genetic disorder caused by different *SMPD1* mutations. Historically, ASMD has been classified as Niemann-Pick disease (NPD) types A (NPD A) and B (NPD B). NPD A is associated with a uniformly devastating disease course, with rapidly progressing psychomotor degeneration, leading to death typically by the age of 3 years, most often from respiratory failure. In contrast, the clinical phenotype and life expectancy of patients with NPD B may vary widely. Almost all patients have hepatosplenomegaly and an atherogenic lipid profile, and most patients have interstitial lung disease with progressive impairment of pulmonary function and hematologic abnormalities including cytopenias. Other common clinical manifestations include liver dysfunction, heart disease, skeletal abnormalities and growth delays. Some patients with ASMD who survive beyond early childhood have intermediate phenotypes (variant NPD B) characterized by combinations of non-neurologic and mild to severe neurologic symptoms. The physical and psychosocial burden of illness in patients with NPD B is substantial. Common symptoms include shortness of breath, joint or limb pain, abdominal pain, bleeding and bruising. The disease often leads to chronic fatigue, limited physical or social activity and difficulties in performing daily activities or work. Many patients die before or in early adulthood, often from pneumonia/respiratory failure or liver failure. Available treatments are limited to symptom management and supportive care. An enzyme replacement therapy currently in clinical development is expected to be the first treatment addressing the underlying pathology of the disease. Early diagnosis and appropriate management are essential for reducing the risk of complications. While knowledge about ASMD is evolving, more evidence about ASMD and the natural history across the disease spectrum is needed, to improve disease recognition, timely diagnosis and appropriate disease management.

## Background

Acid sphingomyelinase (ASM) deficiency (ASMD), a rare lysosomal storage disease, is an autosomal recessive genetic disorder caused by mutations in the *SMPD1* gene [1, 2]. Historically, ASMD has been classified as Niemann-Pick disease type A (NPD A) and Niemann-Pick disease type B (NPD B). NPD A patients have a relatively uniform natural history characterized by severe progressive neurodegeneration in the first year and death typically by 3 years of age [3, 4]. NPD B has a variable disease course and is associated with a broad spectrum of disease severity and manifestations [5]. The term ‘intermediate form’ or ‘variant NPD B’ has also been used to characterize the phenotypes in patients who survive early childhood, and have combinations of non-neurological and also mild-to-severe neurological manifestations [4, 6–8]. Importantly, ASMD represents a disease with a continuum of severity that may not be well defined by the historic NPD A and B classification. In the past, ASMD has also been erroneously grouped with NPD type C (NPD C), a lysosomal storage disorder that is genetically distinct from ASMD, but shares some clinical features (for example, spleen enlargement) [9–11]. Currently, the management of ASMD is limited to symptomatic treatment and supportive care [9]. More evidence is required about ASMD and its natural history across the disease spectrum, in order to raise awareness of the disease among healthcare professionals and to inform clinical decision-making. Accordingly, the objective of this review was to collate and synthesize information available from the published literature on the clinical manifestations and natural history of ASMD, and burden of illness, to help facilitate disease awareness and to support disease recognition, appropriate diagnosis, and patient care.

## Disease overview

### Pathogenesis

ASMD, also known as NPD types A (NPD A) and B (NPD B), is a rare autosomal recessive lysosomal storage disorder caused by mutations^1^ in the ASM-encoding gene *SMPD1* [1, 2]. Insufficient ASM activity results in the abnormal accumulation of the primary ASM substrate, sphingomyelin, and other metabolically-related lipids in cells of the monocyte-macrophage system and other cell types such as hepatocytes. These substrates can build up over time, causing progressive cell and tissue damage and impairment of the functioning of multiple organs. The clinical phenotype of ASMD is highly variable with respect to type and severity of clinical manifestations and may include functional impairment of multiple organs. The severity of ASMD-associated clinical manifestations is influenced by the type of *SMPD1* mutation and appears to reflect the level of residual ASM activity [4]. Pathologic analyses typically reveal large lipid-laden cells, so-called “foam cells”, in the liver, spleen, lymph nodes, adrenal cortex, lungs and/or bone marrow [12]. Infants with severe psychomotor degeneration have profound structural changes in the brain, associated with neuronal cell loss in cerebral and cerebellar cortices, gliosis, demyelination and infiltration of foam cells [12].

### Classification of subpopulations

Patients with ASMD have been categorized historically as NPD A and NPD B based on disease severity and the presence or absence of neurologic symptoms. Patients designated as NPD A have a relatively uniform natural history characterized by severe progressive neurodegeneration in the first year of life and death typically by 3 years of age [3, 4]. In contrast, NPD B has a variable disease course and is associated with a broad spectrum of disease severity and manifestations. Historically, NPD B referred to a heterogeneous population of patients with predominantly chronic progressive visceral manifestations of ASMD [5] and no or only minor neurologic signs and symptoms. Based on evidence presented in the last decades, it has become increasingly apparent that many patients with NPD B also may have significant neurologic symptoms, including progressive neurologic impairment during childhood [4, 6–8]. To distinguish patients who have some neurologic manifestations of ASMD (but do not display the classic NPD A phenotype) from those with a classic non-neurologic NPD B phenotype, they have been variably classified in the literature as variant NPD B, NPD A/B, intermediate ASMD or intermediate NPD [4, 6–8]. Thus, ASMD represents a disease with a continuum of severity that may not be well defined by the historic NPD A and B classification (Table 1). A further source of confusion is the historic grouping of ASMD with NPD type C (NPD C), a lysosomal storage disorder that is distinct from ASMD in genetic, pathologic and prognostic aspects, but shares some clinical features (e.g., spleen enlargement) with ASMD [9–11]. A more comprehensive system of ASMD patient stratification based on molecular, clinical and/or prognostic criteria would be valuable. For the purpose of this review, NPD B generally refers to all patients not classified as NPD A, including those with intermediate phenotypes.

**Table 1:** Classification of patients with ASMD based on historical terms

NPD type A	Intermediate NPD, variant NPD B	NPD type B
Phenotype: Infantile onset of severe neurodegeneration with progressive psychomotor deterioration Natural history: uniform Prognosis: death typically by 3 years of age	Phenotype: NPD B phenotype but also progressive neurologic findings including ataxia, variable degrees of developmental delay and peripheral neuropathy Natural history: variable Prognosis: patients live past early childhood, sometimes into adulthood	Phenotype: Chronic progressive multi-system disease with no or little neurologic involvement Natural history: variable Prognosis: variable (survival until the second to seventh decade of life)

*ASMD* acid sphingomyelinase deficiency, *NPD* Niemann-Pick disease

### Incidence

Reliable estimates of ASMD incidence worldwide are currently lacking. Although ASMD is a pan-ethnic genetic disease, many mutations are private and others are found preferentially in specific ethnic groups [13–20], which may contribute to differences in disease phenotype. The diversity of mutations and frequency of private mutations make it difficult to conduct widespread genetic carrier screening, which can be further complicated by the presence of *SMPD1* sequence variations that do not result in loss of ASM function [21].

Another impediment to obtaining precise estimates of ASMD incidence is the potential for underdiagnosis due to lack of disease awareness. Although some estimates based on actual diagnoses place the incidence of ASMD at approximately 0.5 per 100,000 births [22, 23], estimates extrapolated from the results of carrier screening suggest that the true incidence may be higher in select populations. For example, NPD A has a particularly high prevalence in the Ashkenazi Jewish community, where three different mutations account for >90% of *SMPD1* mutant alleles. Systematic genetic carrier screening for these three mutations in Ashkenazi Jewish adults suggests a carrier frequency of 1 in 80–100, which would extrapolate to an estimated two to three cases per 100,000 births in this population [2]. Similarly, estimates for the Chilean population based on the allele frequency of a single common *SMPD1* mutation associated with NPD B suggest a rate of approximately two cases per 100,000 births, which is higher than the rate suggested by confirmed diagnoses [24].

### Diagnosis

Due to the rarity of ASMD and the heterogeneity of its manifestations, ASMD diagnosis may be missed at initial presentation or occur only at advanced stages of disease progression. ASMD should be suspected in patients with hepatosplenomegaly, developmental delay and/or cherry-red maculae (also described as “perifoveal white patch” [25, 26]), interstitial lung disease, hyperlipidemia characterized by low high-density lipoprotein (HDL) cholesterol and/or thrombocytopenia. In general, the diagnosis of ASMD cannot be based solely on clinical presentation but has to be confirmed by biochemical and/or molecular genetic testing to distinguish ASMD from diseases with similar manifestations, such as Gaucher disease. ASM enzymatic activity can be reliably measured in peripheral blood lymphocytes or cultured skin fibroblasts [12]. The diagnosis of ASMD also can be established by detection of pathogenic *SMPD1* variants through genetic testing. For patients from populations with common known *SMPD1* mutations (e.g., individuals of Ashkenazi Jewish background), targeted analysis for defined pathogenic mutations can be performed, with full-length sequence analysis of *SMPD1* only required if the presence of a mutation in both alleles is not confirmed by other means. In populations in which common mutations do not exist, full-length sequence analysis should always be performed.

Newborn screening for ASMD is feasible by testing the enzymatic activity in dried blood spots [27, 28], and genotyping of *SMPD1* variants, if necessary, can be accomplished with the use of the same dried blood spots. Enzymatic and molecular analysis in noncultivated chorionic villi also is feasible and permits rapid and accurate prenatal diagnosis of ASMD [10]. Genetic carrier screening and prenatal diagnosis of ASMD for couples known to be at risk are available in the Ashkenazi Jewish community due to the high prevalence of a limited number of specific *SMPD1* mutations [10, 29].

A detailed discussion of the diagnosis of ASMD is beyond the scope of this paper. However, a consensus recommendation on a diagnostic guideline for ASMD, which also provides an overview of the relevant differential diagnoses, is available [30].

## Disease manifestations and natural history

Our current understanding of the natural history and overall clinical burden of ASMD is based on single case reports, small case series and a limited number of relatively larger observational studies. A literature search in PubMed since 2004 identified 18 studies that described the natural history and/or the clinical manifestations of patients with ASMD (Table 2 [3–8, 20, 24, 31–40]). However, these studies were often limited in size, ranging from 10 to 103 patients. Therefore, given the genetic and phenotypic heterogeneity of ASMD, caution has to be exercised when generalizing specific observations. Similarly, our current knowledge of the disease progression and causes of death associated with ASMD is based on few studies [3–5, 31, 32, 36].

**Table 2:** Observational studies of ASMD clinical burden since 2004

Reference	Study type	Patients	Objective/Evaluation	Main findings
Lidove et al. 2016 [45]	Retrospective	NPD B (*N* = 28 adults)	Clinical phenotype, laboratory tests	High frequency of MGUS
Cassiman et al. 2016 [31]	Retrospective	NPD B/variant NPD B (*N* = 85)	Cause of death, morbidity	Overall leading causes of death were respiratory failure and liver failure
Acuña et al. 2015 [24]	Retrospective	NPD B: *SMPD1* variant p.(Ala359Asp)	Epidemiology, phenotype	Moderate to severe NPD B with normal cognitive and psychomotor development
McGovern et al. 2013 [32]	Prospective	NPD B, *N* = 103; age 1–72 years	Morbidity, survival, cause of death	NPD B is a life-threatening disorder with morbidity and mortality, especially in children
Wasserstein et al. 2013 [33]	Prospective	NPD B, *N* = 46 (20 children, 26 adults)	Skeletal manifestation (comparative analysis with healthy controls)	Significant association between reduced bone marrow mineral density and increased splenomegaly
Zhang et al. 2013 [20]	Retrospective	ASMD, *N* = 27 (8 NPD A, 4 intermediate, 15 NPD B)	Genotype, phenotype	Comparatively high incidence of NPD A in the Chinese population
Hollak et al. 2012 [4]	Retrospective/ prospective	ASMD, *N* = 25 (4 severe [NPD A], 6 intermediate, 15 attenuated [NPD B])	Clinical phenotype	In NPD B patients, pulmonary disease is the most debilitating clinical feature
Thurberg et al. 2012 [34]^a^	Phase 1 trial (rhASM), (baseline data)	Adults (18–65 years) with ASMD, *N* = 17	Liver and skin pathology	Liver fibrosis in almost all patients. Variable sphingomyelin accumulation; high sphingomyelin accumulation associated with liver enlargement
Henderson et al. 2009 [35]	Prospective qualitative case study	*N* = 17; 8 patients (16–43 years old) with NPD B, 9 parents	Psychosocial burden of disease	Limited physical activity and social isolation and peer rejection are major stressors, particularly for patients 10–16 years
McGovern et al. 2008 [36]	Prospective cross-sectional survey	NPD B, *n* = 59	Suitable endpoints for future clinical trials, (clinical assessments, imaging, QoL [CHQ-PF50, SF-36], laboratory tests)	NPD B patients have multi-system involvement and clinical variable phenotypes. Almost all had splenomegaly, hepatomegaly and interstitial lung disease. Common symptoms: bleeding (49%), pulmonary infections (42%), shortness of breath (42%) and joint/limb pain (39%); low platelets, abnormal lipid values and liver function tests. Delayed growth in adolescence. Mild decrease in QoL with standard instruments
Guillemot et al. 2007 [37]	Retrospective	*N* = 13, 2–9 years old 1 NPD A, 10 NPD B, 2 other (NPD C)	Lung disease	All patients had signs of interstitial lung disease, 1 patient died of respiratory failure, 5 required long-term oxygen therapy
Mihaylova et al. 2007 [6]	Prospective	Intermediate NPD, *N* = 20, 7 months to 35 years old	Phenotype/genotype relationship	Variable neural involvement in patients with intermediate NP and identical genetic background
McGovern et al. 2006 [3]	Prospective longitudinal	NPD A, 10 patients (3–6 months at study entry)	NPD A natural history	All infants had severely impaired cognitive and motor development, cherry-red spots; median survival from diagnosis was 21 months; cause of death was respiratory failure (9 patients) and complications from bleeding (1 patient)
Mendelson et al. 2006 [38]	Prospective	NPD B, *N* = 53	Pulmonary findings	Interstitial lung disease was present in most patients; there was no quantitative correlation between imaging findings and lung function
Wasserstein et al. 2006 [8]	Prospective	NPD B/intermediate NPD, *N* = 64	Prevalence of neurologic disease	10/64 patients had mild hypotonia or hyporeflexia; 5/64 patients had significant progressive neurologic abnormalities including cognitive impairment
Pavlů-Pereira et al. 2005 [7]	Retrospective	ASMD, *N* = 25 (5 NPD A, 4 NPD B, 16 intermediate ASMD)	Phenotype	Description of an intermediate phenotype with overt, borderline or subclinical neurologic symptoms of neuronopathy
McGovern et al. *J Pediatr* 2004 [39]	Prospective	Children with ASMD, *N* = 40 (10 NPD A; 30 NPD B)	Lipid abnormalities	All children had lipid abnormalities including low HDL, high LDL and/or high TG
McGovern et al. *Ophthalmology* 2004 [40]	Prospective	NPD B, *N* = 45 (3–65 years)	Ocular manifestations	15/45 patients had macular stigmata with no evidence of neurodegeneration
Wasserstein et al. 2004 [5]	Prospective longitudinal	NPD B, *N* = 29 (2–64 years at study entry)	NPD B natural history	The natural history of NPD B is characterized by hepatosplenomegaly with progressive hypersplenism, worsening atherogenic lipid profile, gradual deterioration in pulmonary function and stable liver dysfunction

*ASMD* acid sphingomyelinase deficiency, *CHQ-PF50* Child Health Questionnaire – Parental Form 50 for pediatric patients, *HDL* high-density lipoprotein, *LDL* low-density lipoprotein, *MGUS* monoclonal gammopathy of unknown significance, *NPD* Niemann-Pick disease, *Qol* quality of life, *rhASM* recombinant human ASM, *SF-36* Short-Form 36, *TG* triglycerides

^a^Reports baseline observational data from a phase 1 clinical trial

### Manifestations and natural history of NPD A

NPD A is the most severe form of ASMD. Neurologic findings dominate the NPD A phenotype, which is fairly homogeneous in terms of natural history, life expectancy and causes of death [3, 4]. In one study conducted in the United States, natural history data were reported for 10 infants with NPD A, all of whom presented with hepatosplenomegaly at 2 – 4 months of age [3]. Neurologic symptoms were first detected at a median age of 7 months, and by 10 months all infants had developmental arrest followed by rapidly progressing neurodegeneration with deterioration of behavioral, language and gross and fine motor skills. All patients showed progressive hypotonia with loss of deep tendon reflexes, whereas cranial nerve function remained largely intact. Macular cherry-red spots were detectable in all infants by 12 months. Two patients had hydrocephalus, and magnetic resonance imaging performed in three infants revealed delayed myelination in all three patients, widening of the anterior horn of the left ventricle in two patients and an arachnoid cyst in one patient [3]. Histopathologic brain analyses from infants with NPD A typically show characteristic lesions in the cerebellum and to a lesser extent in the cerebrum, with swollen, vacuolated ganglion cells, severe myelin deficiency and accumulation of foam cells and lipid-laden glial cells in the brain and perivascular connective tissue [12].

A typical clinical feature observed in the 10 infants with NPD A described above was irritability beginning around the age of 12 months, with severe sleep disturbance often accompanied by hours-long periods of crying [3]. These symptoms are likely related to the underlying neurologic dysfunction. Failure to thrive was compounded by insufficient intake of calories due to worsening hypotonia, weakened suck and often severe gastrointestinal symptoms. All infants developed progressive respiratory symptoms with frequent respiratory infections secondary to aspiration, and nine of 10 infants eventually died of respiratory failure. The infants also had abnormal laboratory values, including elevated liver enzymes, low HDL cholesterol and progressive decrease in hemoglobin values and platelet counts; one patient died of complications from bleeding. The median time from diagnosis to death was 21 months, with all patients succumbing at or before the age of 3 years [3]. A recent study of the disease spectrum and natural history in 25 patients with ASMD in the Netherlands and Belgium included four infants with NPD A [4]. Similar to the findings in the US cohort, these patients were all diagnosed in the first year of life and had early onset of rapidly progressing deterioration of psychomotor function [4].

### Manifestations and natural history of NPD B

#### Overview

Unlike the fairly homogeneous NPD A phenotype, patients with NPD B have extensive phenotypic heterogeneity, including a wide range of disease manifestations and severity levels and variable rates of disease progression [5]. A prospective, cross-sectional survey of 59 patients (7–65 years of age) from the United States (*n* = 26), Brazil (*n* = 13), Italy (*n* = 8), France (*n* = 7) and Germany (*n* = 5) provided valuable insight in the spectrum of NPD B disease manifestations [36]. For each patient, medical history, physical examinations, assessments of cardiorespiratory function, clinical laboratory data, liver and spleen volumes, radiographic assessments of the lungs and bone age and quality-of-life assessments were obtained according to a standardized protocol. The most common symptoms at initial presentation among these patients were splenomegaly (78%) and hepatomegaly (73%), whereas the most common historical complaints were bleeding (49%, including recurrent epistaxis in 29%), shortness of breath (42%), pulmonary infections (42%), joint and/or limb pain (39%), bruising (27%), headaches (24%), diarrhea (20%) and bone fractures (19%). Clinical laboratory studies revealed that patients commonly had thrombocytopenia, low HDL cholesterol and elevated levels of low-density lipoprotein (LDL) cholesterol, very low-density lipoprotein cholesterol and triglycerides. Elevated serum chitotriosidase and abnormal liver function tests also were noted. Several patients had cardiac disease, including one patient with coronary artery disease who required triple bypass surgery twice within 10 years. Of interest, the degree of splenomegaly correlated with hepatomegaly, growth, lipid profile and hematologic parameters in this study. However, spleen volume showed only weak negative correlations with measures of pulmonary function, including percentage of predicted diffusing capacity of the lung for carbon monoxide (DL_CO_; *r* = −0.306, *p* = 0.052) and percentage of predicted forced vital capacity (*r* = −0.346, *p* = 0.015), and did not correlate with exercise tolerance as measured by the 6-min walk test distance (*r* = −0.260, *p* = 0.075) [36].

Similar clinical findings were documented in a Dutch and Belgian study of 25 patients with ASMD [4]. Of the 21 non-NPD A patients, all but one splenectomized patient had splenomegaly and 19 had hepatomegaly. Thirteen of 16 patients available for evaluation had interstitial lung disease with a variable degree of functional impairment based on pulmonary function testing. In addition, six of 18 patients had slight anemia, 15 of 18 patients had platelet counts <150 × 10^9^/L and most had low HDL cholesterol. Thus, as expected from the underlying pathophysiology, ASMD is associated with multi-organ disease in most patients.

##### Manifestations of NPD B

**Fig. 1:**
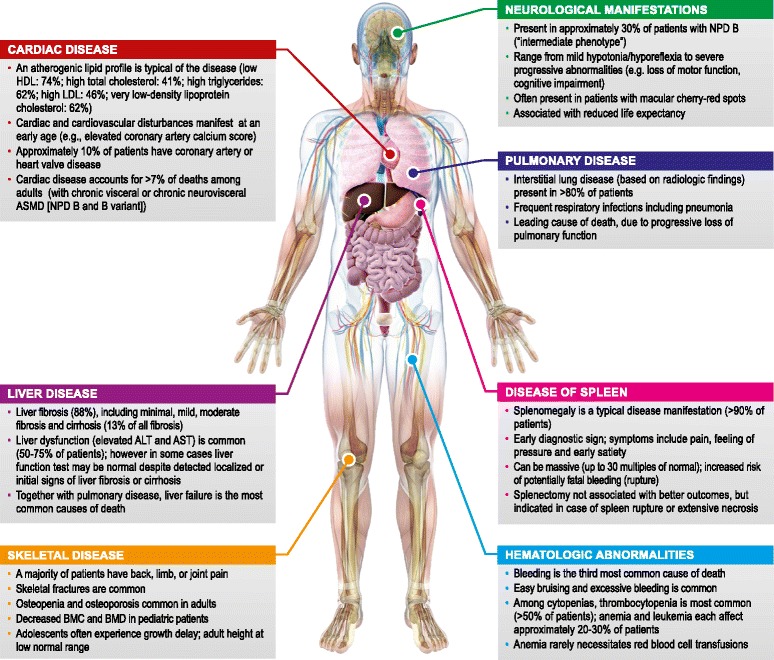
ASMD manifestations in patients with NPD B, in the currently published literature. *ALT* alanine amino transferases; *ASMD* acid sphingomyelinase deficiency; *AST* aspartate amino transferase; *BMC* bone mineral content; BMD bone mineral density; *NPD* Niemann-Pick disease

Typical findings by organ class are discussed in detail below and summarized in Fig. 1.

##### Splenomegaly

Splenomegaly is one of the most common disease manifestations of NPD B [4, 5, 36] and often the first obvious sign of the disease. Evidence from histological spleen samples obtained after surgery, suggests that splenomegaly is essentially the result of infiltration by lipid-laden macrophages [12]. The spleen size can be massive and, as discussed above, may be a surrogate marker of disease severity because of its correlation with other disease parameters [36]. In the cross-sectional study of 59 pediatric and adult patients with NPD B (7 – 65 years of age), splenic volume ranged from 3.1 to 27.3 multiples of normal, with 85% of the patients having spleen volumes greater than 5 multiples of normal [36]. Large spleen size was associated with increased incidence of bleeding and bruising; however, although splenomegaly may be associated with secondary hypersplenism, the study found no significant correlation between spleen volume and platelet count [36].

##### Pulmonary disease

Most patients in the cross-sectional study [36] of 59 patients with NPD B (90%) as well as most patients in a prospective study [38] of 53 patients with NPD B 7 – 65 years of age (90%) had evidence of interstitial lung disease by chest radiography and high-resolution computed tomography. However, the prospective study [38] did not find a strong correlation between radiologic findings and the results of pulmonary function tests. Some patients with markedly abnormal imaging findings had only mild to moderate impairment of gas exchange, and some patients with marked gas exchange impairment (DL_CO_ <60% of predicted value) had only mildly abnormal imaging findings [38]. Therefore, imaging studies are not sufficient in the evaluation of pulmonary disease in NPD B and must be interpreted in conjunction with functional testing and the clinical status of the patient. Among patients with functional pulmonary disease, the most common findings are low forced vital capacity and DL_CO_, which are consistent with restrictive lung disease and impaired gas exchange secondary to interstitial lung disease. A natural history study of 29 pediatric and adult patients with NPD B documented slow progression of pulmonary disease with small mean annual changes in all measures of pulmonary function [5]. Slow progression of pulmonary disease also has been reported by others [37]. Overall, respiratory disease is one of the most common manifestations and a leading cause of death in patients with NPD B [31, 32].

##### Liver disease

In a study of morbidity and mortality in 103 patients with NPD B carried out at a single center between 1992 and 2012, six patients had fulminant liver failure, and liver biopsies from three additional patients showed evidence of cirrhosis [32]. Two of the patients with liver failure received successful orthotopic liver transplants at 12 and 25 years of age, whereas three of four patients who did not receive a transplant died from liver failure [32].

In the cross-sectional study of 59 patients with NPD B, alanine aminotransferase (ALT) and aspartate aminotransferase (AST) were elevated in 51% of the patients, and total bilirubin was elevated in 33% of patients [36]. In the natural history study of 29 patients with NPD B (2–64 years of age at study entry), liver dysfunction was common, with 75% of patients having elevated ALT and 65% having elevated AST at the initial visit. However, overall no statistically significant changes in liver enzyme values over time were noted [5]. One patient developed hepatic dysfunction approximately 2 years after his last visit and subsequently died of liver failure [5]. Another study, a systematic analysis of liver biopsies from 17 adult patients with NPD B revealed the presence of liver fibrosis in 15 patients. Two of the patients (39 and 40 years of age) had frank cirrhosis in the absence of any clinical symptoms of liver failure [34]. A recent case report of a patient who died at the age of 31 years from refractory encephalopathy related to cirrhosis and hepatic failure, further illustrates that hepatic tests may be normal despite evidence of extensive cirrhosis in liver biopsies [41].

In a series of 13 Chilean pediatric patients homozygous for the *SMPD1* p.(Ala359Asp) (A359D) mutation associated with moderate to severe NPD B, five (38.5%) patients developed clinically relevant liver disease due to progressive cirrhosis [24]. Notably, all five patients had sustained approximately four-fold increases in ALT and AST. Three of these patients succumbed to liver failure in childhood and the other two received liver transplants [24]. In summary, available evidence suggests that the prevalence of liver disease is high and that the incidence of clinically significant liver disease varies in different patient populations, and may be as high as 40% in some NPD B subpopulations. In addition to respiratory disease, liver failure is the most common cause of death in patients with NPD B [31].

##### Skeletal disease

According to medical history, 19% of the patients in the cross-sectional study had suffered one or more bone fractures [36]. A subsequent detailed examination of the skeleton was carried out in 20 pediatric and 26 adult patients with NPD B, including Dual X-ray Absorptiometry scans to measure bone mineral content (BMC) and bone mineral density (BMD) [33]. Five (25%) pediatric and 14 (53%) adult patients had a history of skeletal fracture. Additionally, 12 (60%) pediatric patients had experienced back and leg pain and 15 (58%) adult patients had experienced back pain. Pediatric patients had significant decreases in adjusted mean BMC and BMD at the lumbar spine, hip and femoral neck compared with a cohort of healthy age-matched subjects. In addition, most adults with NPD B had osteopenia or osteoporosis at one or more sites according to the World Health Organization classification of BMD [33]. These findings demonstrate that skeletal involvement is a common feature of ASMD.

##### Cardiac disease and hyperlipidemia

In the cross-sectional study, electrocardiogram abnormalities were present in 16 of 58 (28%) patients evaluated [36]. These included sinus bradycardia (*n* = 6), left ventricular hypertrophy (*n* = 4) and conduction abnormalities (*n* = 6). Two-dimensional echocardiograms showed abnormalities in 29 (50%) patients, most commonly mild mitral valve regurgitation. However, two patients had previously undiagnosed moderate to severe aortic regurgitation and one patient had pulmonary hypertension [36].

Most patients in the cross-sectional study [36] had an atherogenic lipid profile characterized by low HDL cholesterol (74% of 58 patients; mean 26 mg/dL), high total cholesterol (41% of 58 patients), high triglycerides (62% of 58 patients) and high LDL (46% of 57 patients) and very low-density lipoprotein (62% of 34 patients) cholesterol compared with age- and gender-matched control subjects. The mean total cholesterol/HDL cholesterol ratio (*n* = 58) of 10.3 was 2.3 times the upper limit of normal [36]. In a study of pediatric patients with ASMD, including 10 with NPD A and 30 with NPD B, all patients displayed abnormal fasting lipid profiles [39]. Mean levels of total, HDL and LDL cholesterol, and triglycerides were abnormal in both NPD A and NPD B patients, with no substantial differences between the two groups. Furthermore, electron beam tomography of the coronary arteries performed in 18 NPD B patients revealed positive calcium scores (range 1.4–34.5) in 10 patients, which in patients <18 years of age suggests the presence of early atherosclerosis. Age and coronary artery calcium scores in this pediatric population were strongly correlated (*r* = 0.79, *p* < 0.0001), and the highest score (34.5; >95th percentile for age and sex) was present in a 18 year old female [39]. Thus, cardiac and cardiovascular involvement is part of the ASMD phenotype at an early age. Consistent with these findings, a systematic evaluation of morbidity and mortality in 103 patients with NPD B further showed that 9% of the patients had coronary artery or heart valve disease [32].

##### Hematologic findings

The cross-sectional study documented bleeding episodes in 49% of the 59 patients enrolled [36]. The most common bleeding event was recurrent epistaxis in 29% of patients, with two patients requiring repeated cauterizations. Significant bleeding events in one patient each included subdural hematoma, hematemesis, hemoptysis, hemothorax, excessive bleeding after tonsillectomy and adenoidectomy resulting in a blood transfusion, menorrhagia and uterine bleeding that required a hysterectomy [36]. Recurrent epistaxis also was a clinical characteristic of all 13 Chilean patients with homozygous *SMPD1* A359D mutations in a recent study [24]. Presumably, these bleeding episodes are related in part to thrombocytopenia, which in the cross-sectional study was present in 53% of patients at the time of evaluation and the most common hematologic abnormality. In comparison, anemia and leukopenia were present in 26% and 21% of patients, respectively [36].

##### Growth

Growth data collected in the cross-sectional study [36] revealed that most patients with NPD B had below average height and weight, with mean z scores (based on normative growth data from the Centers for Disease Control) of −1.3 (range −4.88 – 2.14) and −0.77 (range −5.22 – 1.80), respectively. Growth delay was most pronounced in adolescents (mean height z score −2.60, in patients 13–17 years of age), who also had delayed bone age that corresponded with delayed puberty. However, most adults (≥18 years of age) had heights in the low normal range (mean height z score −0.58), suggesting that a period of catch-up growth occurs in late adolescence and/or early adulthood [36]. Thus, although short stature is a cause of concern for adolescent patients with NPD B, final adult heights appear to approach normal values in most patients.

### Neurologic manifestations of variant NPD B

As noted above, there are patients with ASMD who do not present with the classic NPD A phenotype but have variable neurologic symptoms ranging from mild hypotonia or hyporeflexia to severe progressive neurologic abnormalities such as loss of motor function and mental deterioration [4, 6–8, 32, 42, 43]. In these patients, the onset of neurologic symptoms is later in life than in patients with NPD A and are not characterized by rapid progression. In a report of 25 Czech and Slovak patients with ASMD who did not demonstrate the classic NPD A phenotype, 16 (64%) displayed a spectrum of neurologic symptoms [7]. In this case series, 12 of the 16 patients had the Q292K mutation in homoallelic or heteroallelic form, and 10 of those had a protracted neurovisceral phenotype [7]. In a prospective US study of 64 patients with NPD B who underwent detailed neurologic and ophthalmologic examinations, 19 (30%) were found to have neurologic abnormalities, again suggesting that patients with a phenotype that includes neurologic manifestations constitute a significant proportion of ASMD patients in addition to the NPD A patient population [8]. The most common abnormalities were mild hypotonia and/or hyporeflexia, which were found in 10 patients. Five of the 19 patients, who had been diagnosed between the ages of 15 months and 5 years, had progressive neurologic abnormalities. Unlike infants with NPD A, these five patients reached their normal developmental milestones at least during the first 2 years of life. Varying from patient to patient, the onset of neurologic difficulties, which included cognitive impairment (mental deterioration or expressive language delay) and/or other moderate to severe neurologic symptoms, occurred at 2 – 7 years of age [8]. Patients with macular cherry-red spots often but not always had neurologic abnormalities [8, 40].

Of note, a patient with NPD B who was diagnosed at age 59 and died at age 60 from severe restrictive lung disease, also had Parkinson’s disease [4]. Although the pathogenic relationship between ASMD and Parkinson’s disease in this patient is unknown, it recently has been suggested that at least some *SMPD1* mutations are associated with an increased risk of Parkinson’s disease [44].

A recent paper analyzing the phenotype of 28 adult French patients with ASMD found a peripheral neuropathy (*n* = 3), depression requiring anti-depressant therapies (*n* = 3), and psychosis (*n* = 1) [45].

#### Natural history of NPD B

In a single-center longitudinal US study, 29 patients with NPD B had serial evaluations over a 10-years period to evaluate disease progression [5]. All patients with intact spleens had splenomegaly, with spleen volumes ranging from 4.5 to 27.3 multiples of normal. In addition, all but one patient had hepatomegaly. Progressive decreases in platelet and leukocyte count over time were documented, as was an overall worsening of the atherogenic lipid profiles. Similarly, all measurements of pulmonary function showed a gradual but slow deterioration over time, whereas liver dysfunction was generally characterized by stable elevation of liver enzymes and bilirubin [5]. The natural disease course of NPD-B patients was evaluated in a recent study among patients from the Netherlands and Belgium [4]. Of the twenty-one non-NPD A patients, only six patients with the attenuated form of NPD-B were included in the prospective natural history study; four patients had a detailed follow-up up through to 6 years. A detailed assessment of these patients revealed stable disease parameters, with slow progression of pulmonary disease [4]. In the study of 13 pediatric Chilean patients with homozygous *SMPD1* A359D mutations, five developed fulminant liver failure over an average follow-up period of 10 years [24]. The different observations in these study populations reflect the heterogeneity of natural history in patients with NPD B and may be related to the underlying *SMPD1* genotypes. In both the US [5] and European [4] studies, a variety of *SMPD1* mutations were present, whereas the study with Chilean patients [24] included the same genotype.

## Mortality

As noted above, mortality in NPD A is most frequently due to respiratory failure by the age of 3 years [3]. In contrast, mortality data for NPD B are too heterogeneous and limited to allow for the construction of survival curves. Available evidence suggests that survival among patients with NPD B varies significantly, consistent with their phenotypic heterogeneity. Although many patients do not survive into adulthood, some have reached their fifth or sixth decade of life [32]. Results of an analysis of morbidity and mortality in 103 patients with NPD B that also included patients with significant neurologic manifestations were recently reported [32]. A total of 18 patients died during the 20-years study period, at a median age of 15.5 years (range 1–72 years). Twelve deaths occurred in patients aged ≤21 years, yielding a mortality rate of 19% in this age group. Overall, the most common causes of death were pneumonia/respiratory failure (five patients), acute liver failure (three patients), bleeding complications (three patients) and complications from bone marrow transplants (three patients); other causes of death (each in one patient) included multi-organ failure, heart failure and liver cancer [32].

A more recent study evaluated disease-related morbidities and the primary causes of death in 85 patients: with chronic visceral ASMD (NPD B excluding variant NPD B, *n* = 58) and chronic neurovisceral ASMD (variant NPD B, *n* = 27), including 78 patients who had died and seven patients who had received liver transplants because of terminal liver disease [31]. Overall, the most common causes of death were respiratory disease (27.7% of patients) and liver disease (27.7%), followed by bleeding (9.6%) and cardiac disease (7.2%). The median age at the time of death was 23.5 and 8 years for patients with chronic visceral and chronic neurovisceral ASMD, respectively. Among patients with chronic neurovisceral ASMD (31.8%), neurodegenerative disease progression was a leading cause of death (23.1%), along with respiratory disease (23.1%) and liver disease (19.2%) [31].

## Genotype-phenotype relationship

More than 180 different *SMPD1* mutations have been identified to date in patients with ASMD, including missense, nonsense, frameshift mutations and splice variants [46]. Because of the recessive nature of these mutations, ASMD generally requires the inheritance of two mutant alleles. However, *SMPD1* is a preferentially paternally imprinted gene [47]. Consequently, heterozygous carriers of a maternal *SMPD1* mutation may show mild forms of ASMD [47]. Predicting ASMD natural history based on genetic data is challenging because of the multitude of possible allelic combinations and because many mutations are private [15]. Nevertheless, extensive mutational analyses in ASMD patient communities worldwide have provided some insight into important genotype-phenotype relationships [13, 20]. For example, NPD A in the Ashkenazi Jewish community is predominantly associated with three *SMPD1* mutations (R496L, L302P and fsP330) [48–50]. Because of the obvious prognostic and diagnostic significance of these mutations, carrier screening is an important tool for genetic counseling in this community. In Chile, a single *SMPD1* mutation (A359D) may account for most cases of NPD B and appears to be associated with a typical clinical phenotype with normal cognitive and psychomotor development but moderate to severe visceral disease manifestations, including a high incidence of clinically significant liver disease [24]. The R610del mutation is the most common mutation in patients with ASMD, with a particularly high prevalence among patients of North African origin [51]. It is associated with an attenuated NPD B phenotype and considered “neuroprotective”, as even pairing with a null allele or another deleterious neuronopathic allele (e.g., R496L) did not lead to progressive neurodegenerative disease [8, 13]. However, some patients heterozygous for the R610del allele have mild hypotonia or hyporeflexia [8]. 677delT and R608 alleles were found to be associated with severe NPD A in Israeli Arabs and with NPD B in northern Africa, respectively [51, 52].

Evidence from two studies suggests that the Q292K mutation is strongly associated with neurologic involvement. Specifically, the Q292K mutation was present in heteroallelic or homoallelic form in 10 of 12 Czech and Slovak patients with progressive neurologic disease [7], and three of four patients with a Q292K mutation (homoallelic or paired with a null allele) had progressive neurologic symptoms in a US study [8]. In contrast, in a group of 20 patients with an intermediate phenotype who were homozygous for a unique ancestral *SMPD1* mutation (W391G), neurologic manifestations were generally highly diverse but similar among relatives [6]. This raises the possibility that, in addition to the *SMPD1* mutation profile, other genetic factors may influence the ASMD phenotype.

## Quality of life, psychosocial and economic burden

Information about the impact of ASMD on health-related quality of life (QoL) and the psychosocial burden of ASMD has to be derived from case reports, as very few studies have attempted to address these topics. Owing to the rarity and heterogeneity of ASMD, there is a lack of robust quantitative data regarding the impact of the disease on patients’ and caregivers’ QoL. This deficiency is compounded by the lack of validated disease-specific instruments to evaluate QoL in patients with ASMD, and the limitations of generic QoL instruments in providing useful quantitative data [36]. The economic impact of ASMD on the affected individuals and their families has not been studied.

The devastating burden of NPD A for the affected children and their families in terms of physical and emotional impact is self-evident and reflected in testimonials by parents of children with the disease [53]. Before or by the age of 1 year, infants with NPD A show increased signs of irritability and discomfort, including inability to sleep, prolonged periods of crying and frequent vomiting [3]. Around-the-clock care for their infants has a profound negative effect on caregivers’ QoL. In addition, physical and occupational therapy for children with NPD A as well as frequent hospitalization resulting from respiratory infections can be expected to be associated with a significant economic burden for the patients’ families [3].

A single study [36] assessed QoL in a limited number of adult and pediatric patients with NPD B using generic QoL instruments (i.e., the Child Health Questionnaire – Parental Form 50 for pediatric patients [CHQ-PF50] and the Short-Form 36 [SF-36] for adults). Four of 10 pediatric patients had CHQ-PF50 subscale scores suggesting diminished QoL associated with physical functioning, mental health, general health perceptions and/or emotional well-being. Using the SF-36, the study found only mild decreases in the score in adult patients compared with the US general population [36]. The apparent inconsistency of these QoL findings with the known clinical symptom burden of the patients (see above) [36] illustrates the need for validated age-adequate ASMD-specific QoL instruments.

The human experience and psychosocial impact of the disease has been evaluated in a small number of adolescent and adult patients with NPD B (*n* = 8) and the parents (*n* = 9) of the adolescent patients [35]. The study identified psychosocial problems based on interviews with patients and caregivers, and six patients (three adults and three adolescents) provided quantitative data for a measure of psychosocial development. Limited physical activity and social isolation were identified as major psychosocial stressors in all patients. In pediatric patients, social isolation was often linked to exclusion from physical activities such as roughhousing, soccer and wrestling, which increase the risk for splenic rupture and are discouraged. In addition, juvenile patients often suffered from peer rejection owing to enlarged abdomen from hepatosplenomegaly, growth and developmental delay. For adults, chronic fatigue and lack of energy appeared to be major factors leading to social isolation [35]. In the quantitative test of psychosocial development, participants’ scores indicated resolution conflicts related to intimacy, isolation, ego integrity and despair, consistent with anxiety and feelings of missed opportunities in life, particularly in the area of relationships. Patients and parents also consistently expressed frustration about the lack of treatment and paucity of medical information [35].

Parents and caregivers often have difficulties maintaining their emotional and mental health while caring for their children with a life-threatening disease. In addition, afflicted families may face extreme financial burden given that the multifaceted disease course in many pediatric and adult patients is associated with repeated emergency hospitalization, particularly for bleeding events and respiratory complications, chronic need for supportive care such as oxygen therapy or psychosocial care, and progressive mental or physical disability that may require expensive special therapies [32].

Overall, there is a lack of quantitative and qualitative data on QoL and the impact of the disease on patients and families. In addition, there is limited information as to the specific disease manifestations that are most bothersome to patients with ASMD and how they affect their daily functioning and QoL.

## Therapeutic options

There are currently no curative therapies for ASMD. Some experimental approaches including bone marrow transplantation, total lung lavage and amniotic cell transplant have been attempted but do not have a favorable benefit/risk ratio owing to their uncertain impact on short- and long-term disease outcomes and considerable risk of complications [2]. Splenectomy may be required in cases of extensive spleen necrosis with loss of function [54] or rupture [55, 56]; however, it is generally not recommended as it may exacerbate pulmonary disease [36]. Thus, management of patients with ASMD is limited to supportive care and palliation, which requires a multidisciplinary approach [9]. Physical and occupational therapy in infants with NPD A may be beneficial but should be directed with realistic goals. Patients with progressive pulmonary disease may require chronic oxygen therapy and modification of their daily activities. Vaccination against influenza and *Streptococcus pneumoniae* species should be considered to minimize the risk of pneumonia. Standard lipid-lowering agents are indicated for the treatment of ASMD-associated lipid abnormalities in adult patients. Although some patients have undergone growth hormone treatment for short stature with consequent acceleration of linear growth [57], most patients have a period of catch-up growth with continued acquisition of height into their twenties. To date, no treatment approach has been reported to positively affect low bone density, and there are no effective treatments to reduce hepatosplenomegaly. Because of low platelet count and risk of hematoma or bleeding, sports involving strong physical contact, such as soccer and wrestling, must be avoided. Many patients rarely become anemic enough to require transfusions. However, patients with clinically significant cytopenia and a history of excessive bleeding may require multiple blood transfusions.

Enzyme replacement therapy (ERT) with recombinant human ASM is a potentially disease-modifying therapeutic approach currently in clinical development for the treatment of ASMD. ERT represents a known mechanism of action that has been used successfully in other lysosomal storage disorders [58]. Results of a 26-weeks phase 1b study in five adult patients with NPD B established initial proof of concept for the safety and efficacy of recombinant human ASM in this patient group, including reductions in sphingomyelin storage seen in liver biopsies, spleen and liver volumes and serum chitotriosidase activity, and improvements in infiltrative lung disease, lipid profiles, platelet counts and QoL assessments [59]. A phase 1/2 clinical trial in pediatric patients and a phase 2/3 trial in adult patients with ASMD are ongoing [60–62].

## Conclusions

ASMD is a rare genetic, progressive life-threatening disease with highly variable severity and disease course. NPD A has a devastating clinical course associated with onset of rapidly progressing psychomotor degeneration often shortly after the first 6 months of age. Children typically die from respiratory failure by the age of 3 years. NPD B is a chronic progressive disease, representing a spectrum of phenotypes with varying severity levels, progression rate and prognosis. NPD B is associated with a substantial burden for many patients, caused by the profoundly negative impact of its clinical manifestations on physical, mental and psychosocial well-being. However, published data regarding the impact of the disease on QoL and daily functioning are limited, mainly owing to the rarity and heterogeneity of the disease and the lack of adequate disease-specific instruments to measure QoL. Given the wide spectrum of NPD B phenotypes, including intermediate forms characterized by mild to severe neurologic defects in addition to visceral, skeletal and hematologic ASMD manifestations, a refinement of the ASMD classification system is needed. A more precise description of ASMD subpopulations may improve disease recognition at initial presentation and thus lead to more timely diagnoses. Better characterization and delineation of different ASMD subpopulation will require additional studies able to improve our current understanding of the disease and the factors that influence disease phenotype, natural history and prognosis.

## Abbreviations

ALT: Alanine aminotransferase
ASM: Acid sphingomyelinase
ASMD: Acid sphingomyelinase deficiency
AST: Aspartate aminotransferase
BMC: Bone mineral content
BMD: Bone mineral density
CHQ-P50: Child health questionnaire – parental form 50
ERT: Enzyme replacement therapy
HDL: High-density lipoprotein
LDL: Low-density lipoprotein
NPD A and B: Niemann-Pick disease types A and B
QoL: Quality of life
SF-36: Short-form 36

## Footnotes

1 There are currently two numbering systems for *SMPD1* mutations as a result of two reference sequences (GenBank Accession Numbers NM_000543.4 and M81780.1) that differ by a length polymorphism within the signal peptide region. Reference to mutations should refer to the appropriate reference sequence for clarity; in this paper, the NM_000543.4 sequence was used.
